# Supplementary material for: Operation of spinal sensorimotor circuits controlling phase durations during tied-belt and split-belt locomotion after a lateral thoracic hemisection
Source: bioRxiv. 2024 Dec 5:2024.09.10.612376. Originally published 2024 Sep 14. Preprint. [Version 2] doi: 10.1101/2024.09.10.612376 (PMC11419089; doi:10.1101/2024.09.10.612376)
Supplement: Supplement 1 [file NIHPP2024.09.10.612376v2-supplement-1.pdf]

bioRxiv preprint doi: https://doi.org/10.1101/2024.09.10.612376; this version posted December 5, 2024. The copyright holder for this preprint (which was not certified by peer review) is the author/funder, who has granted bioRxiv a license to display the preprint in perpetuity. It is made available under aCC-BY 4.0 International license.

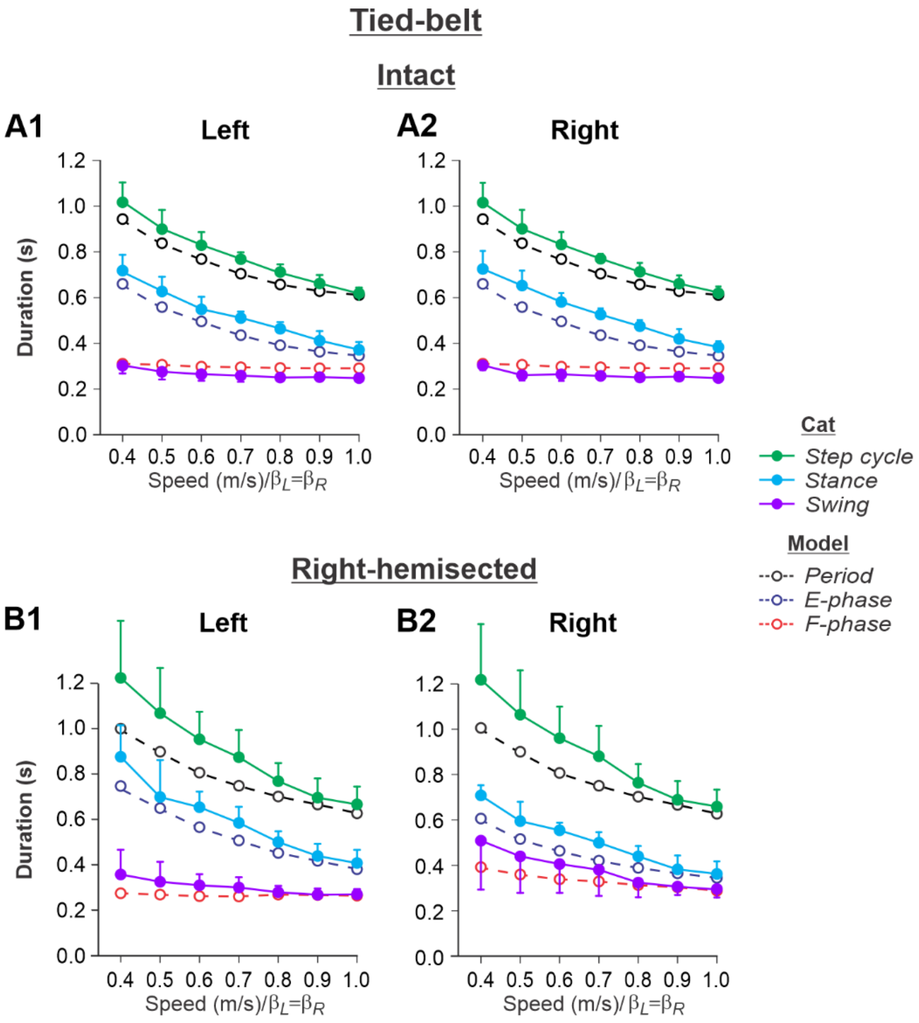

Figure 5 - figure supplement 1. Comparison of simulated and experimental data during tied-belt locomotion. The figure overlays simulated and experimental data from Figures 2 and 5, respectively, in the intact (**A1, A2**) and hemisected (**B1, B2**) states for the left and right hindlimbs.

bioRxiv preprint doi: https://doi.org/10.1101/2024.09.10.612376; this version posted December 5, 2024. The copyright holder for this preprint (which was not certified by peer review) is the author/funder, who has granted bioRxiv a license to display the preprint in perpetuity. It is made available under aCC-BY 4.0 International license.

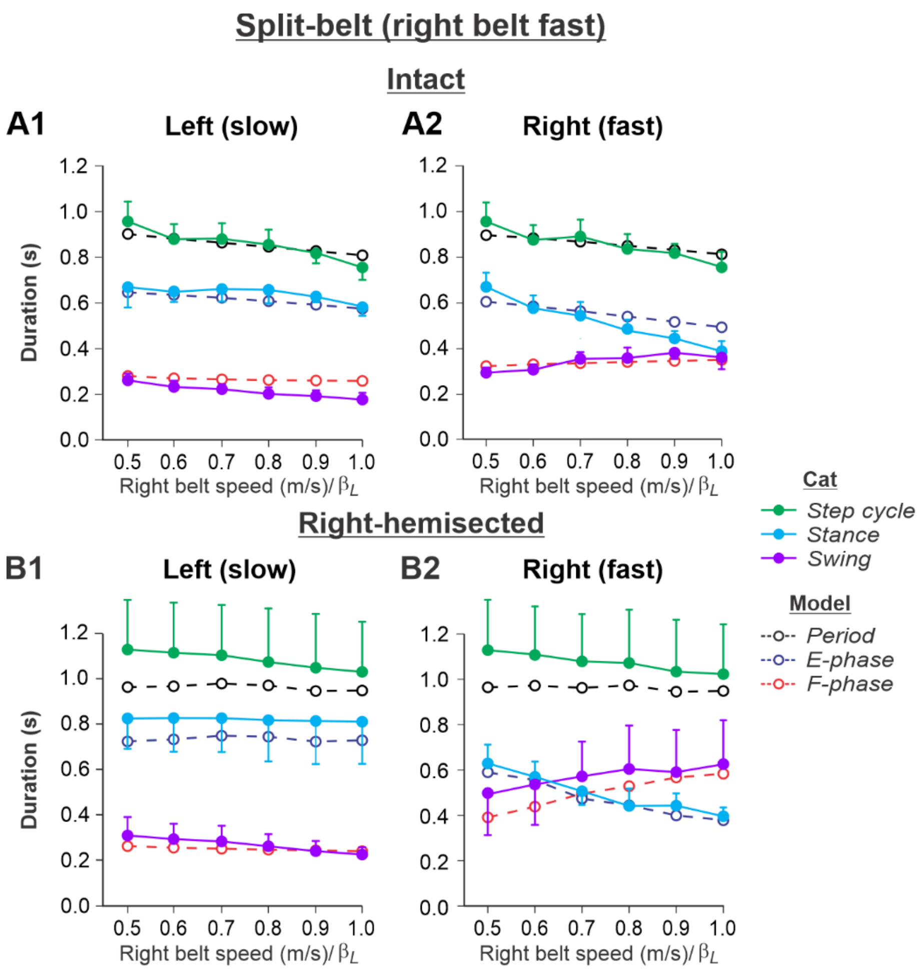

Figure 6 – figure supplement 1. Comparison of simulated and experimental data during split-belt locomotion in the *Left slow/Right fast condition*. The figure overlays simulated and experimental data from Figures 3 and 6, respectively, in the intact (**A1, A2**) and hemisected (**B1, B2**) states for the left and right hindlimbs.

bioRxiv preprint doi: https://doi.org/10.1101/2024.09.10.612376; this version posted December 5, 2024. The copyright holder for this preprint (which was not certified by peer review) is the author/funder, who has granted bioRxiv a license to display the preprint in perpetuity. It is made available under aCC-BY 4.0 International license.

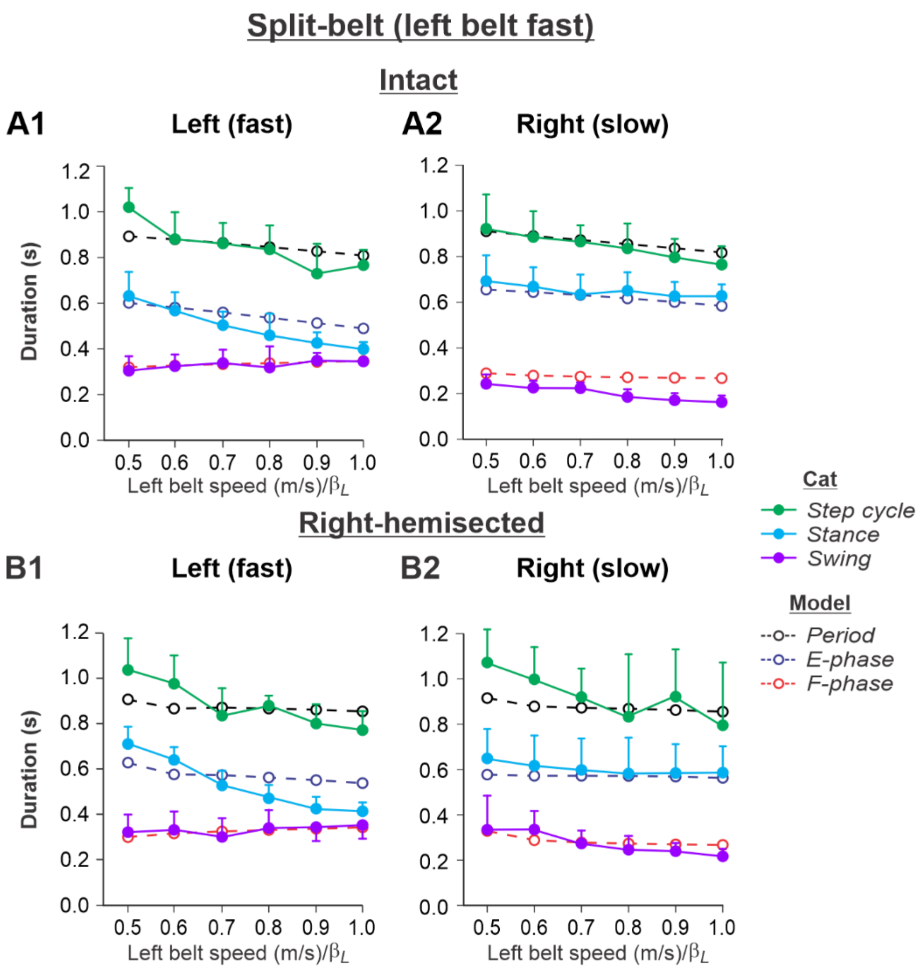

Figure 7 – figure supplement 1. Comparison of simulated and experimental data during split-belt locomotion in the *Left fast/Right slow condition.* The figure overlays simulated and experimental data from Figures 4 and 7, respectively, in the intact (**A1, A2**) and hemisected (**B1, B2**) states for the left and right hindlimbs.
